# Frontomaxillary Facial Angle Measurement in Screening for Trisomy 18 at 11 + 0 to 13 + 6 Weeks of Pregnancy: A Double-Centre Study

**DOI:** 10.1155/2013/168302

**Published:** 2013-10-01

**Authors:** Bartosz Czuba, Wojciech Cnota, Agata Wloch, Piotr Wegrzyn, Krzysztof Sodowski, Miroslaw Wielgos, Dariusz Borowski

**Affiliations:** ^1^Teaching Department of Obstetrics and Gynecology in Ruda Slaska, Medical University of Silesia, Ulica Lipa 2, 41-703 Ruda Slaska, Poland; ^2^Teaching Department of Obstetrics and Gynecology of the Medical University of Warsaw, Plac Starynkiewicza 1/3, 02-015 Warsaw, Poland

## Abstract

*Objective*. The aim of this study was to evaluate the effectiveness of prenatal screening for trisomy 18 with the use of the frontomaxillary facial angle (FMF angle) measurement. *Material and Methods*. The study involved 1751 singleton pregnancies at 11–13 + 6 weeks, examined between 2007 and 2011. Serum PAPP-A and free beta-hCG levels were assessed, and crown-rump length, nuchal translucency, and FMF angle were measured in all patients. 1350 fetuses with known follow-up were included in the final analysis. *Results*. Highly significant (*P* < 0.01) negative correlation between the CRL and the FMF angle was found. There were 30 fetuses with trisomy 18. FMF angle was highly significantly larger (*P* < 0.0001) in fetuses with trisomy 18 as compared to chromosomally normal fetuses. Two models of first trimester screening were compared: Model 1 based on maternal age, NT, and first trimester biochemistry test (DR 80–85% and FPR 0.3–0.6%), and Model 2 = Model 1 + FMF angle measurement (DR 87.3–93.3% and FPR 0.8–1.3%). *Conclusions*. The use of FMF angle measurement increases the effectiveness of the screening for trisomy 18. Introduction of the FMF angle as an independent marker for fetal trisomy 18 risk requires further prospective research in large populations.

## 1. Introduction

Highly effective prenatal screening for fetal chromosomal defects is based on analysis of the maternal age, ultrasound measurement of the nuchal translucency between the 11 + 0 and 13 + 6 weeks of pregnancy, and the first trimester biochemistry test (free beta-hCG and PAPP-A) [1, 2]. Analysis of those factors allows achieving high detection rate, including detection of trisomy 18—detection rate (DR) of approximately 80–90% and false positive rate (FPR) below 5% [2]. According to the Polish Gynecological Society Guidelines the risk limit of 1 : 300 calculated with a certified program for trisomy 21 is a cut-off for invasive diagnostics [3]. The value determines simultaneously the FPR factor [1, 2, 4]. Assuming 1 : 300 ratio as the cut-off point determining high risk of occurrence of trisomy 18, DR is 97%, and FPR is 3.1% [5].

Both the literature reports and clinical practice indicate a possible value of additional, trisomy ultrasound markers for increasing the efficacy of screening with reduction of number of invasive procedures [6–8]. Studies on applicability of the frontomaxillary facial angle (FMF angle) as a marker of trisomy 21, 18, and 13 were conducted [9–11], and normal values for FMF angle in euploid fetuses were determined [12]. Fetuses with trisomy 18 had significantly higher value of the FMF angle compared to euploid fetuses. Inclusion of FMF angle measurement in prenatal screening caused increase of the DR to 94%, with the false positive results rate maintained at the level of 5%, compared to the combined screening. Acceptance of the DR at the level of 92% caused simultaneous reduction in number of indications for invasive diagnostics to the level of 3% [5]. Those values were reported for trisomy 21. The available literature suggests that the FMF angle measurement could also be a useful ultrasound marker for trisomy 18 [10].

The purpose of the study was to assess the efficacy of prenatal screening for trisomy 18 using the FMF angle measurement, depending on cut-off point.

## 2. Material and Methods

The study involved 1751 singleton pregnancies in 2007–2011. In all cases CRL, NT, and FMF angle were measured, and the first trimester biochemistry test was performed. 1350 fetuses, with known follow-up (normal karyotype or trisomy 18), were qualified for the final analysis. We performed 145 amniocenteses for fetal karyotyping. In the remaining cases trisomy 18 phenotype was excluded during clinical examination by an experienced neonatologist. Trisomy 18 is characterized by a broad spectrum of dysmorphological features and congenital defects. Cases of other chromosomal defects and congenital malformations were excluded from the study.

**Figure 1:**
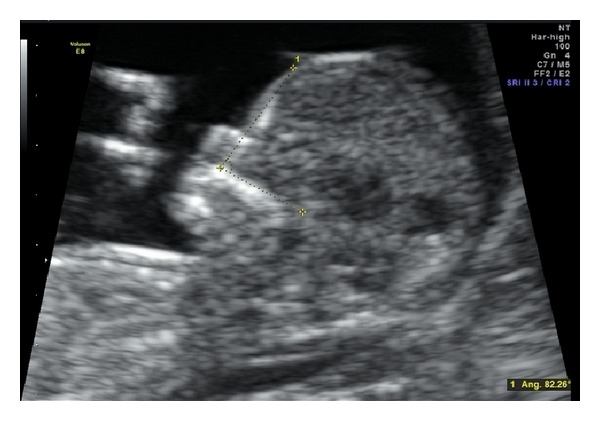
Measurement of the FMF angle.

1320 normal cases were used to create normal range and calculate 95 percentile for four arbitrarily created intervals based on CRL. Maternal age at the moment of the assessment was recorded, and the crown rump length (CRL) of fetuses was measured. In all cases the NT and FMF angle was measured according to principles of the Fetal Medicine Foundation, London (FMF). FMF angle is formed between the superior surface of the maxilla and the frontal bone and was measured in the midsagittal section of the fetal face at 11 + 0 to 13 + 6 weeks [12, 13] (Figure 1). All scanning physicians had valid FMF certificates. Ultrasound examinations were performed transabdominally using the Voluson Expert 730 and the Voluson Expert E8 systems (General Electrics). 

The literature data indicate a very important role of an appropriate fetal head cross-section for reliable and repeatable measurements [14]. For an inexperienced operator the number of examinations necessary for achievement of high quality and repeatability of the FMF angle measurement ranged between 90 and 140, whereas an operator experienced in nuchal translucency measurement becomes proficient in the FMF angle measurement after performing 40 examinations [15].

First trimester biochemistry tests were performed using the Delfia Express equipment (Perkin Elmer) on at least 2 mL serum samples. All data were entered to the Astraia database (Astraia Software GmbH) for determination of the risk of trisomy 18. Patients were divided into two groups: fetuses with trisomy 18 and chromosomally normal fetuses. The statistical analyses were performed with the PQStat suite, v. 1.4.2.324. The test probability at the level of *P* < 0.05 was regarded significant, and *P* < 0.01 was regarded highly significant. Spearman's rank correlation and Pearson correlation analysis were also performed. Values of the FMF angle in healthy fetuses and fetuses with trisomy 18 were compared using the Mann-Whitney *U* test.

For assessment of efficacy of the prenatal screening for trisomy 18 with the FMF angle measurement (as an addition to the T18 risk analysis based on maternal age, first trimester biochemistry test result, and NT measurement) detection rates and false positive results rates were calculated depending on the risk cut-off value for trisomy 18 (four risk groups were determined: 1 : 300, 1 : 200, 1 : 100, and 1 : 50). In relation to the CRL value, a 95 percentile was determined for the FMF angle. Cross tabulation to create contingency tables; chi-squared test and Fischer's exact test were used in analyzing models.

## 3. Results

**Figure 2:**
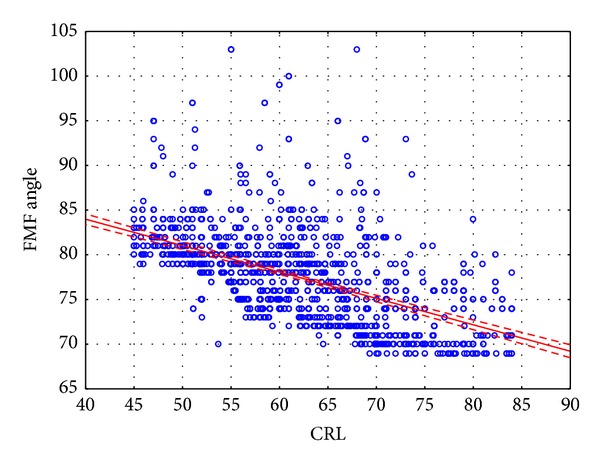
Correlation between CRL and FMF angle.

Median maternal age was 31 years (range 14–45 years; SD = 5.2 years). The median FMF angle was 77° (range 69°–103°; SD = 5.1°). The median CRL measurement was 62.2 mm (range 45–84 mm; SD = 9.6 mm). The median NT measurement was 1.5 mm (range of 0.5 to 12.7 mm; SD = 0.8 mm). No significant correlation was found between the maternal age and the FMF angle. A highly significant (*P* < 0.01) negative correlation was found between the CRL value and the FMF angle (Figure 2). Values of the 95 percentile were calculated for the FMF angle for four previously defined CRL ranges. For CRL between 45.0 and 55.9 mm 95th centile equaled 89°, and for CRL 56.0–65.9 mm it was 85°, for CRL 66.0–75.9 it was 84°, and for CRL 76.0–84.0 mm it was 78°. 

**Figure 3:**
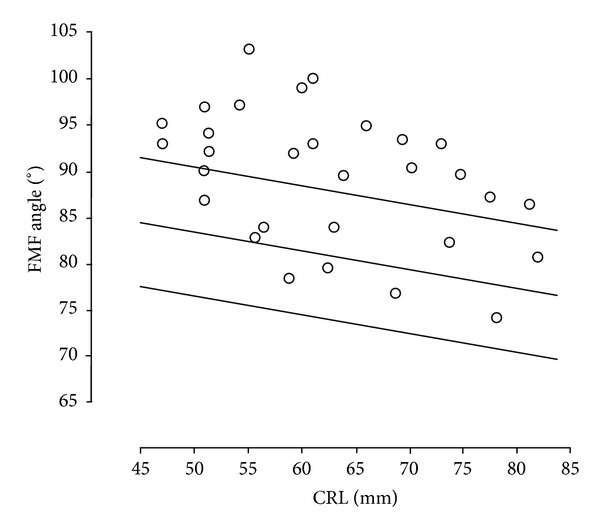
Values of FMF angle in trisomy 18 fetuses (median, 95th and 5th centile).

Thirty cases of trisomy 18 were diagnosed. Values of the FMF angle above the 95 percentile were observed in 19 fetuses with trisomy 18 (63.3%) (Figure 3). FMF angle was highly significantly larger (*P* < 0.0001) in fetuses with trisomy 18 as compared to chromosomally normal fetuses (Table 1). 

In the study group the detection rate and the false positive results rate were calculated for four risk levels as cut-off points for the model based on the maternal age, NT measurement, and first trimester biochemistry test, Model 1 (Table 2), and for the screening model based on the maternal age, NT measurement, first trimester biochemistry test, and measurement of the frontomaxillary facial angle, Model 2 (Table 3).

## 4. Discussion

Facial dysmorphic features are characteristic traits in many aneuploidies, including trisomy 18. During the 1st trimester of pregnancy, flat shape of the face can be seen on ultrasound. The shape of the fetal profile depends on the value of the FMF angle. 

Borenstein et al. showed that the values of the FMF angle in euploid fetuses range from 84.3° to 76.5°, depending on the CRL value (45–84 mm), and a negative correlation was demonstrated between the CRL and the FMF angle. [12]. We also demonstrated a negative correlation between the CRL and the FMF angle. Our observations indicate that the FMF angle values in euploid fetuses range between 103° and 69°. 

According to Borenstein et al. data, the value of the FMF angle in fetuses with trisomy 18 were over the 95 percentile in approximately 60% of examined fetuses [10]. In our study, the angle measurement above 95 percentile was observed in approximately 63% of trisomy 18 cases. Similarly to Borenstein et al. we demonstrated no correlation between the value of the FMF angle and the NT value, which makes the FMF angle measurement suitable as an additional marker [10].

Kagan et al. studied efficacy of prenatal trisomy 18 screening based on maternal age, NT measurement, and value of the first trimester biochemistry test [5]. Their results indicate increasing DR in the range of 88–97% with increasing FPR in the range of 0.1–5%, with application of different mathematic models for trisomy 18 and trisomy 21. Our results (Model 1—without the FMF angle measurement) based on trisomy 18 prenatal screening show DR in the range of 80–85%, with FPR from 0.3 to 0.5%, depending on the cut-off point. Addition of the FMF angle as a marker of trisomy 18 (Model 2) caused an increase of DR from 85 to 93.3%, with FPR ranging from 0.8 to 1.3%, respectively, depending on the cut-off point. It is notable that in that set of data, both screening models show increase of DR, with the change of the cut-off point, with only minor increase of FPR. 

Sonek et al. showed that FMF angle measurement as an additional marker of trisomy 21 increases detectability of the syndrome, with simultaneous reduction in number of invasive procedures [9].

Lachmann et al. found correlation between the frontomaxillary facial angle and occurrence of defects of the central nervous system. The authors observed significantly lower values of the FMF angle (below the 5 percentile) in 90% of fetuses with rachischisis and open myelomeningocele at 11 + 0 to 13 + 6 weeks [16]. In our study we found no cases of rachischisis in the group of fetuses with trisomy 18. Studies on fetuses with trisomy 13 demonstrated that in those cases the FMF angle is increased only in cases with holoprosencephaly [11]. 

Depending on the cut-off point and accepted rate of false positive results, hence invasive procedures rate, the obtained increase of DR for trisomy 18 with low FPR suggests possible applicability of measurement of the FMF angle in routine screening at 11 + 0 to 13 + 6 weeks. Larger prospective studies are required to test the usefulness and cost effectiveness of implementation of the FMF angle in first trimester screening for trisomy 18.

## 5. Conclusions

1. The use of frontomaxillary facial angle (FMF angle) measurement for prenatal screening of trisomy 18 increases the effectiveness of the screening.
2. Introduction of the FMF angle as an independent marker for fetal trisomy 18 risk requires further prospective research in large populations.

## Figures and Tables

**Table 1:** FMF angle in chromosomally normal and in trisomy 18 fetuses.

	Minimum (°)	Lower quartile (°)	Median (°)	SD	Upper quartile (°)	Maximum (°)	Mann-Whitney *U* test
Normal karyotype	69.0	74.0	77.0	4.6	80.0	103.0	<0.0001
Trisomy 18	83.0	84.0	94.5	6.4	97.0	103.0	

**Table 2:** Model 1—based on the maternal age, first trimester biochemistry test, and NT measurement, in four predefined trisomy 18 risk cut-off points.

Risk	1 : 300	1 : 200	1 : 100	1 : 50
DR (%)	85	85	83	80
FPR (%)	0.5	0.5	0.4	0.3
PPV (%)	57.8	65.0	73.7	66.7
NPV (%)	99.7	99.7	99.8	99.8
LR+	55.15	73.66	111.06	80.0
LR−	0.14	0.13	0.067	0.067
*P* value	<0.0001	<0.0001	=0.0014	=0.0003

**Table 3:** Model 2—as in Model 1 plus the FMF angle.

Risk	1 : 300	1 : 200	1 : 100	1 : 50
DR (%)	93.3	90.0	87.0	87.3
FPR (%)	1.3	1.1	0.9	0.8
PPV (%)	96.2	96.3	99.8	99.8
NPV (%)	99.2	99.2	99.6	99.6
LR+	425.83	428.58	476.94	476.94
LR−	0.17	0.16	0.07	0.07
*P* value	<0.0001	<0.0001	<0.0001	<0.0001
